# Plumbagin Suppresses α-MSH-Induced Melanogenesis in B16F10 Mouse Melanoma Cells by Inhibiting Tyrosinase Activity

**DOI:** 10.3390/ijms18020320

**Published:** 2017-02-03

**Authors:** Taek-In Oh, Jeong-Mi Yun, Eun-Ji Park, Young-Seon Kim, Yoon-Mi Lee, Ji-Hong Lim

**Affiliations:** 1 Department of Biomedical Chemistry, College of Biomedical and Health Science, Konkuk University, Chungju 380-701, Korea; dk1050@kku.ac.kr (T.-I.O.); dbswjdalgg@naver.com (J.-M.Y.); peunji0503@kku.ac.kr (E.-J.P.); yskim0801@kku.ac.kr (Y.-S.K.); 2 Interdisciplinary Research Center for Health, Konkuk University, Chungju 380-701, Korea; yoonmilee@kku.ac.kr

**Keywords:** plumbagin, melanogenesis, pigmentation, tyrosinase

## Abstract

Recent studies have shown that plumbagin has anti-inflammatory, anti-allergic, antibacterial, and anti-cancer activities; however, it has not yet been shown whether plumbagin suppresses alpha-melanocyte stimulating hormone (α-MSH)-induced melanin synthesis to prevent hyperpigmentation. In this study, we demonstrated that plumbagin significantly suppresses α-MSH-stimulated melanin synthesis in B16F10 mouse melanoma cells. To understand the inhibitory mechanism of plumbagin on melanin synthesis, we performed cellular or cell-free tyrosinase activity assays and analyzed melanogenesis-related gene expression. We demonstrated that plumbagin directly suppresses tyrosinase activity independent of the transcriptional machinery associated with melanogenesis, which includes micropthalmia-associated transcription factor (*MITF*), tyrosinase (*TYR*), and tyrosinase-related protein 1 (*TYRP1*). We also investigated whether plumbagin was toxic to normal human keratinocytes (HaCaT) and lens epithelial cells (B3) that may be injured by using skin-care cosmetics. Surprisingly, lower plumbagin concentrations (0.5–1 μM) effectively inhibited melanin synthesis and tyrosinase activity but do not cause toxicity in keratinocytes, lens epithelial cells, and B16F10 mouse melanoma cells, suggesting that plumbagin is safe for dermal application. Taken together, these results suggest that the inhibitory effect of plumbagin to pigmentation may make it an acceptable and safe component for use in skin-care cosmetic formulations used for skin whitening.

## 1. Introduction

Melanin consists of a group of pigments synthesized in epidermal melanocytes that plays an important role in defending skin against ultraviolet (UV) radiation damage [1]. Abnormal melanogenesis, either increased or decreased production of melanin, is closely associated with a number of skin diseases including melanoma and the pigmentation disorders, such as chloasma and freckles [2,3]. Biosynthesis of melanin is initiated by multiple stimuli including UV irradiation, inflammatory cytokines, and hormonal signaling. Specifically, α-melanocyte stimulating hormone (α-MSH) released from UV-exposed keratinocytes can stimulate melanin biosynthesis in epidermal melanocytes by activating the cAMP-PKA-CREB (cyclic adenosine monophosphate-protein kinase A-cAMP response element binding protein) axis [3]. The activated cAMP-PKA-CREB axis leads to an increase in mRNA encoding micropthalmia-associated transcription factor (MITF). MITF increases the gene expression of tyrosinase (*TYR*), tyrosinase-related protein 2 (*TYRP1*), and tyrosinase-related protein 2 (*TYRP2*) upon α-MSH stimulation in melanocytes [3,4]. In addition, many signaling pathways that control cell growth, including mitogen-activated protein kinases (MAPKs), extracellular response kinase (ERK), and AKT, are essential to melanogenesis by regulating MITF stability and activity [5].

The enzyme tyrosinase, a multifunctional copper-containing oxidase, plays an essential role in melanin biosynthesis by catalyzing the reactions in which l-tyrosine is hydroxylated to l-dihydroxyphenylalanine (l-DOPA) and l-DOPA is oxidized into *o*-quinone (dopaquinone) [5,6]. Therefore, some tyrosinase inhibitors also inhibit melanin biosynthesis, and these include resveratrol, arbutin, and hinokitiol, which have all been used in skin-whitening cosmetic applications [7].

Plumbagin is a simple hydroxyl-naphthoquinone, was first extracted from the roots of the *Plumbago* genus of plants, and has been shown to have remarkable medicinal properties [8]. Anti-inflammatory, anti-cancer, anti-allergic, and antibacterial activities of plumbagin have been reported [8,9]. Indeed, it has been reported that plumbagin inhibits neutrophil activation, angiogenesis, and collagenase expression by suppressing topoisomerase-II, suggesting the use of plumbagin as a potential drug in the treatment of rheumatoid arthritis [10]. In addition, several reports have shown that plumbagin exhibits anti-cancer activity in multiple types of cancer, including breast [11], prostate [12], ovarian [13], lung [14], skin carcinoma [15], and liver cancer [16]. Although it is becoming clear that plumbagin may be useful as a therapeutic intervention in the treatment of various human diseases, the inhibitory effect of plumbagin on melanogenesis linked to hyperpigmentation has never been reported.

In the present study, we evaluated the inhibitory effects of plumbagin on melanogenesis stimulated by α-MSH. Here we show that plumbagin significantly suppresses α-MSH-induced melanin biosynthesis in B16F10 mouse melanoma cells by inhibiting tyrosinase activity but that it does not inhibit MITF-mediated gene expression associated with melanogenesis.

## 2. Results

### 2.1. Chemical Structure and Cytotoxic Effects of Plumbagin in B16F10 Mouse Melanoma Cells

**Figure 1:**
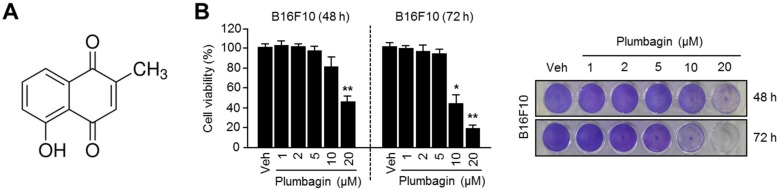
Chemical structure and cytotoxicity of plumbagin. (**A**) Chemical structure of plumbagin; (**B**) toxicity of plumbagin in B16F10 mouse melanoma cells. Cells were incubated with 1, 2, 5, 10, 20 μM of plumbagin for 48 or 72 h. Values (left panel) represent mean ± SD of three independent experiments performed in duplicate; * *p* < 0.05 and ** *p* < 0.01. Crystal violet staining images are shown in the right panel.

Before studying the anti-melanogenic effects of plumbagin, we first assessed its toxicity in melanin-producing B16F10 mouse melanoma cells. The chemical structure of plumbagin is shown in Figure 1A. The results of our cytotoxicity assay wherein plumbagin concentrations less than 5 μM did not affect cell viability in B16F10 cells are shown in Figure 1B.

### 2.2. Plumbagin Suppresses α-MSH-Induced Melanin Synthesis in B16F10 Mouse Melanoma Cells

**Figure 2:**
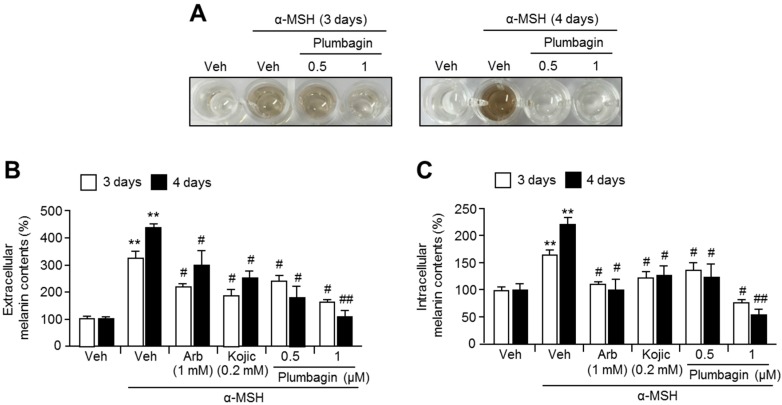
Effects of plumbagin on melanin production in B16F10 mouse melanoma cells. (**A**) Plumbagin suppressed α-MSH-induced melanin production. Cells were pre-incubated in the absence or presence of plumbagin for 1 h, following which α-MSH (0.2 mM) was added and the cells were incubated for 3 or 4 days. Color changes in the cultured medium are shown; (**B**) extracellular and (**C**) intracellular melanin content increased by α-MSH treatment alone and decreased when plumbagin treatment was also given. Cells were pre-incubated with arbutin (1 mM), kojic acid (0.2 mM), or plumbagin (0.5, 1 μM) for 1 h, and then further incubated with α-MSH (0.2 mM) for 3 or 4 days as indicated. Values represent means ± SD of three independent experiments performed in duplicate; # *p* < 0.05, ## *p* < 0.01, and ** *p* < 0.01.

We next investigated the inhibitory effects of plumbagin on α-melanocyte stimulating hormone (α-MSH)-induced melanin synthesis in B16F10 cells. We demonstrated that plumbagin strongly suppresses α-MSH-induced melanin accumulation in a cultured medium of B16F10 cells (Figure 2A). To confirm the inhibitory effect of plumbagin on α-MSH-induced melanin synthesis, we determined the extracellular or intracellular melanin content in the absence or presence of plumbagin in α-MSH-stimulated B16F10 cells. Figure 2B,C show that plumbagin significantly decreases extracellular and intracellular melanin content.

### 2.3. Plumbagin Does Not Regulate α-MSH-Induced Melanogenesis Gene Expression and Signal Transduction Cascades

**Figure 3:**
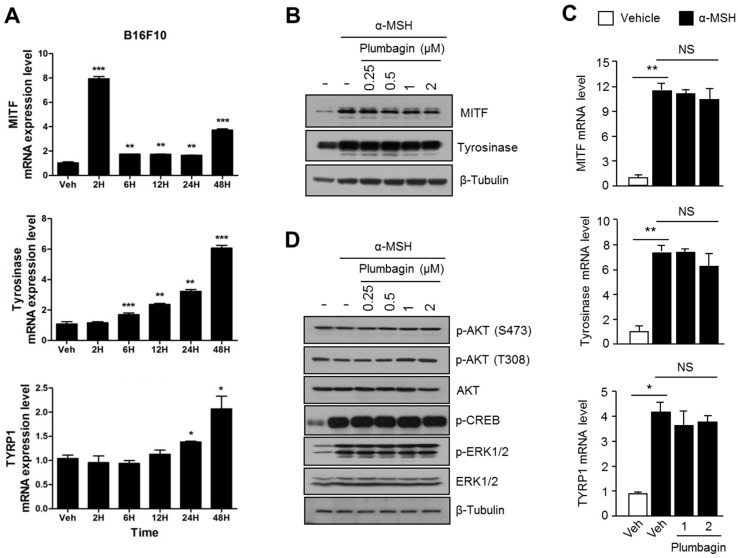
Plumbagin does not affect the transcriptional machinery and signal transduction cascade associated with melanogenesis. (**A**) Determination of time to mRNA expression associated with melanogenesis. Cells were incubated with 0.2 mM of α-MSH for indicated time periods, following which melanogenesis-related gene-specific mRNA expression level was measured. Values represent means ± SD of two independent experiments performed in triplicate; * *p* < 0.05, ** *p* < 0.01, and *** *p* < 0.001; (**B**) effects of plumbagin on MITF and tyrosinase protein expression levels. B16F10 cells pre-incubated with plumbagin (0.25, 0.5, 1, 2 μM) were further incubated with 0.2 mM α-MSH. MITF and tyrosinase protein expression levels were measured via immunoblotting as described in the materials and methods section; (**C**) effect of plumbagin on MITF, TYR, and TYRP1 mRNA expression. B16F10 cells were incubated in the absence or presence of α-MSH and plumbagin (1, 2 μM) for 4 h (MITF mRNA) or 48 h (TYR and TYRP1 mRNA). MITF, TYR, and TYRP1 mRNA expression levels were measured using quantitative RT-PCR. Values represent means ± SD of three independent experiments performed in triplicate; * *p* < 0.05, ** *p* < 0.01, and NS (not significant); (**D**) regulatory effects of plumbagin on signal transduction proteins that participate in melanogenesis. Cells were pre-incubated with plumbagin for 1 h, and cells were then further incubated with α-MSH (0.2 mM) for 3 h. Indicated protein levels were measured via immunoblotting.

Because micropthalmia-associated transcription factor (MITF) is an essential transcription factor that regulates melanogenesis-associated gene expression through the α-MSH-PKA-CREB axis, we investigated whether plumbagin could regulate MITF-mediated gene expression associated with melanogenesis. First, we determined a time point of maximal melanogenesis gene expression for *MITF*, tyrosinase (*TYR*), and tyrosinase-related protein 1 (*TYRP1*) under α-MSH stimulation. *MITF* is strongly upregulated after α-MSH treatment for 2 h (Figure 3A). *TYR* and *TYRP1* were dramatically upregulated after 48 h of α-MSH treatment (Figure 3A). MITF and tyrosinase protein levels increased in response to α-MSH treatment and were not suppressed by the plumbagin treatment (Figure 3B). Consistently, plumbagin did not inhibit *MITF*, *TYR*, and *TYRP1* mRNA levels after α-MSH stimulation, suggesting that plumbagin does not regulate the transcriptional machinery related to melanogenesis gene expression in B16F10 cells (Figure 3C). Because phosphorylation and activation of AKT, ERK1/2, and CREB (major signal transduction cascades that regulate melanogenesis) are required for melanogenesis [3], we further investigated whether plumbagin regulates these melanogenesis-associated signal transduction pathways. Our results, described in Figure 3D, show that plumbagin does not alter AKT, ERK1/2, or CREB signaling after α-MSH treatment.

### 2.4. Plumbagin Inhibits Tyrosinase Activity

**Figure 4:**
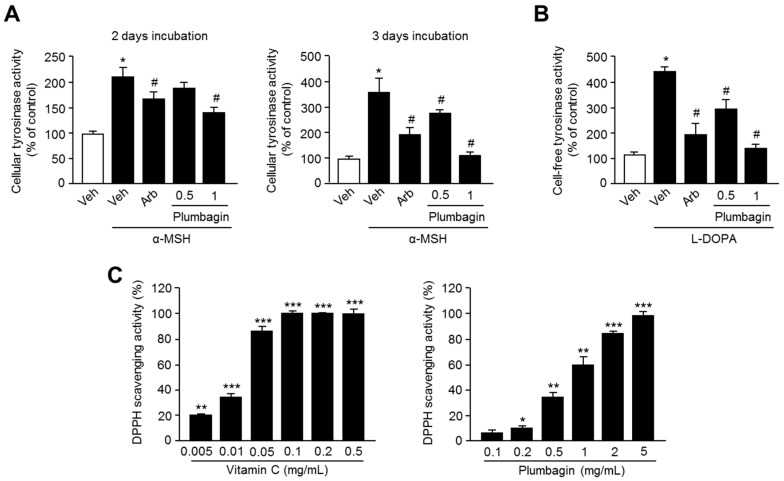
Inhibitory effects of plumbagin on (**A**) cellular tyrosinase activity and (**B**) cell-free tyrosinase activity. Tyrosinase activity was determined by measuring l-DOPA oxidation to dopachrome, and this oxidation of l-DOPA was read using an absorbance reader at 475 nm; (**C**) antioxidants activity of plumbagin. DPPH scavenging activity was examined at indicated concentrations using plumbagin or vitamin C as a positive control. Values represent means ± SD of three independent experiments performed in triplicate; * *p* < 0.05, ** *p* < 0.01, *** *p* < 0.001 and # *p* < 0.05.

Because it is clear that direct or indirect tyrosinase-inhibiting natural products could be useful for the development of skin-whitening cosmetics, we next investigated the inhibitory effect of plumbagin on tyrosinase enzyme activity. In this study, we demonstrated that plumbagin significantly suppresses α-MSH-induced cellular tyrosinase enzymatic activity in B16F10 cells (Figure 4A). To understand whether plumbagin inhibits tyrosinase activity directly or indirectly, cell-free tyrosinase activity was measured. Plumbagin strongly inhibits l-DOPA oxidation activity of mushroom-derived tyrosinase as well, suggesting that plumbagin suppresses melanogenesis by directly inhibiting tyrosinase activity (Figure 4B). In addition, significant elevation in radical scavenging activities using a 1,1-diphenyl-2-picrylhydrazyl (DPPH) assay was observed in samples treated with vitamin C and plumbagin at concentrations ranging from 0.1 to 5 mg/mL. Consequently, 5 mg/mL of plumbagin exhibited a similar effect on radical scavenging activity to those of 0.1 mg/mL of vitamin C (Figure 4C).

### 2.5. Plumbagin Is Not Toxic in Normal Keratinocytes and Lens Epithelial Cells

**Figure 5:**
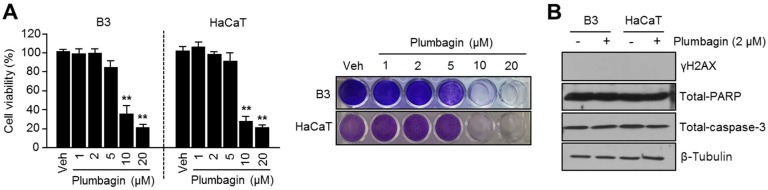
Cytotoxic effects of plumbagin in B3 and HaCaT cells. (**A**) Cytotoxicity of plumbagin in B3 and HaCaT cells. Cells were incubated with various concentrations (1, 2, 5, 10, 20 μM) of plumbagin for 3 days. Values (left panel) represent means ± SD of three independent experiments performed in duplicate; ** *p* < 0.01. Crystal violet staining images were shown (right panel); (**B**) plumbagin does not cause DNA damage and apoptosis in B3 and HaCaT cells. Cells were incubated with or without 2 μM of plumbagin for 3 days, and protein levels were then measured via immunoblotting.

Because cosmetics such as skin cream are for external use and cytotoxicity could cause skin problems, we further investigated whether plumbagin is toxic to normal keratinocytes (HaCaT) and lens epithelial cells (B3) and whether it may be useful for the development of skin-whitening cosmetics. Consequently, we found that lower plumbagin concentrations (1–5 μM) do not cause toxicity in HaCaT and B3 cells, but are effective on inhibition of melanin synthesis (Figure 5A). In addition, plumbagin did not induce DNA damage-related γH2AX and reduce cellular apoptosis-related poly (ADP-ribose) polymerase (PARP) and caspase-3 proteins in HaCaT and B3 cells (Figure 5B), suggesting that plumbagin may be acceptable for use in skin-whitening products without exhibiting any dermal toxicity.

## 3. Discussion

Plumbagin is a plant-derived secondary metabolite and shows several biological functions, including anti-inflammatory, anti-cancer, anti-allergic, and antibacterial activities [8,9]. However, it was not known whether plumbagin had an inhibitory effect on melanogenesis linked to hyperpigmentation and melanoma. In this study, we demonstrated that plumbagin strongly suppresses melanogenesis in B16F10 melanoma cells, and this work could provide an opportunity for developing skin-care cosmetics based on the anti-inflammatory, anti-allergic, antibacterial, and anti-melanogenesis activities of plumbagin.

**Figure 6:**
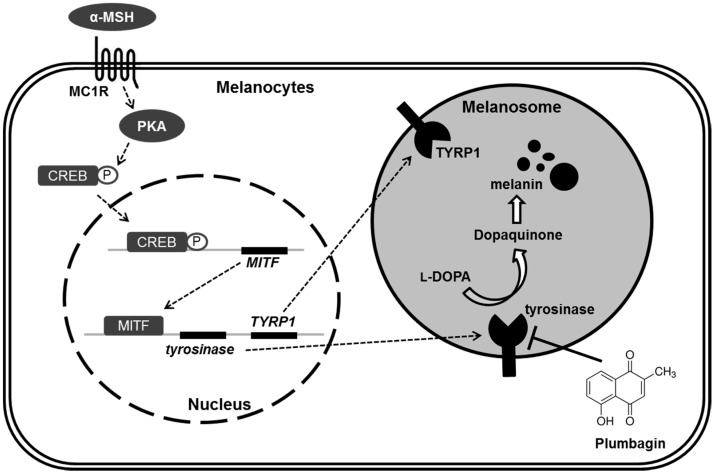
Proposed regulatory mechanism of plumbagin on suppression of melanin synthesis in melanocytes. The dotted line and white arrows indicate stimulating signals. The black T bar indicates inhibitory effect. α-MSH: alpha-melanocyte stimulating hormone; MC1R: melanocortin 1 receptor; PKA: protein kinase A; CREB: cAMP response element binding protein; MITF: micropthalmia-associated transcription factor; TYR: tyrosinase; TYRP1: tyrosinase-related protein 1; l-DOPA: l-3,4-dihydroxyphenylalanine.

Because upregulation of melanogenesis is often observed in malignant melanoma with overexpressed tyrosinase levels in blood as well as tumor tissues, it is apparent that melanogenesis is a potential target for chemotherapy of malignant melanomas [1]. In the present study, we found that plumbagin directly suppresses tyrosinase activity independent of the transcriptional machinery associated with melanogenesis (Figure 6). Therefore, our results suggest that plumbagin may be a potential component of a preventive and therapeutic strategy for the management of malignant melanoma. Indeed, the anti-melanoma, chemosensitizing, and radiosensitizing effects of plumbagin on melanoma therapy have been reported [17,18,19,20,21].

In previous studies dealing with the anti-cancer and anti-inflammatory effects of plumbagin, approximately 5–10 μM of plumbagin (higher than the concentration used in this study) has been used in in vitro models [17,22,23]. Therefore, we investigated the toxic effect of plumbagin in proliferative cells, including normal keratinocytes (HaCaT) and lens epithelial cells (B3), that can be damaged by skin-care cosmetics. Our results demonstrated that lower concentrations, approximately 0.5–1 μM of plumbagin, are not toxic to normal keratinocytes and lens epithelial cells, but are effective enough to reduce tyrosinase activity and melanin synthesis. In addition, we also found that plumbagin more effectively suppresses melanogenesis than 1 mM arbutin or 0.2 mM kojic acid, which are well-known skin-whitening agents. These results suggest that plumbagin is safe for use as a component for developing skin-whitening cosmetics.

In the present study, we studied the inhibitory effects of plumbagin on the tyrosinase enzyme reaction using cellular and cell-free tyrosinase activity assays and based on l-DOPA oxidation. Nevertheless, we did not suggest a precise molecular mechanism as to how plumbagin inhibits tyrosinase activity. This mechanism should be further investigated to determine whether plumbagin directly interacts with tyrosinase and inhibits its oxidase activity or whether it has indirect effects that lead to decreased tyrosinase activity.

## 4. Materials and Methods

### 4.1. Reagents and Antibodies

Plumbagin, α-MSH (M4135), arbutin (A4256), kojic acid (K3125), l-DOPA (333786), and tyrosinase (T3824) purified from mushrooms were purchased from Sigma-Aldrich (St. Louis, MO, USA). Antibodies against MITF (#12590), p-AKT^S473^ (#4060), p-AKT^T308^ (#13038), p-CREB (#9198), p-ERK1/2 (#4370), ERK1/2 (#9102), γH2AX (#9718), PARP (#5625), and caspase-3 (#9665) were purchased from Cell Signaling Technology (Danvers, MA, USA). Tyrosinase (sc-7833), and β-tubulin (sc-9104) was purchased from Santa Cruz Biotechnology (Dallas, TX, USA).

### 4.2. Cell Culture and Cell Viability Assay

B16F10 (mouse melanoma cells), B3 (human lens epithelial cells), and HaCaT (human keratinocyte) cells were cultured in Dulbecco’s modified Eagle’s medium (DMEM) supplemented with 10% fetal bovine serum and 25 mM glucose. The cells were incubated in a humidified atmosphere of 95% air and 5% CO_2_ at 37 °C. To measure viability, cells were incubated with various concentrations of plumbagin dissolved in dimethyl sulfoxide (DMSO) for 48 or 72 h. After incubation, cultured cells were washed with PBS and then incubated with 0.5% crystal violet staining solution for 20 min at room temperature. To measure optical density, stained cells were incubated with 1% sodium dodecyl sulfate (SDS) solution for 15 min at room temperature, and the optical density of each well was then measured at 570 nm (OD_570_) using an absorbance reader (BioTek, Winooski, VT, USA).

### 4.3. Immunoblotting

Cultured cells were lysed using 1% IGEPAL (octylphenoxypolyethoxyethanol), 150 mM NaCl, 50 mM Tris-HCl (pH 7.9), 10 mM NaF, and protease inhibitor cocktail in lysis buffer. Protein samples were separated via sodium dodecyl sulfate-polyacrylamide gel electrophoresis (SDS-PAGE), and the separated proteins were then transferred to a polyvinylidene difluoride (PVDF) membrane (Millipore, Billerica, MA, USA). Membranes were incubated overnight with primary antibodies (1:1000) at 4 °C, and then incubated with HRP-conjugated secondary antibodies (1:10,000) for 1 h at room temperature. Proteins were visualized using an ECL Prime kit (GE healthcare, Pittsburgh, PA, USA).

### 4.4. Measurement of Intracellular and Extracellular Melanin Content

Melanin content was determined and quantified using a previously described method with slight modification [2,24]. For melanin content analysis, B16F10 cells were cultured in phenol red-free DMEM containing 10% fetal bovine serum. B16F10 cells were cultured with α-MSH treatment in the absence or presence of plumbagin for 3 or 4 days. The cultured cells or media were harvested and pellets were dissolved in 1N NaOH containing 10% DMSO at 80 °C for 1 h. The melanin content was measured using an absorbance reader at 475 nm (OD_475_), and melanin content was then normalized to the cellular protein concentration.

### 4.5. RNA Isolation and Quantitative RT-PCR

Total RNA was extracted from B16F10 cells using TRIzol (Invitrogen, Waltham, MA, USA) and 2 µg of total RNA was used for cDNA synthesis by using a high capacity cDNA reverse transcription kit (Applied Biosystems, Waltham, MA, USA). Quantitative PCR was performed using SYBR Green PCR Master Mix (Applied Biosystems). The sequences of the PCR primers (5′–3′) were as follows: TCAAGTTTCCAGAGACGGGT and CATCATCAGCCTGGAATCAA for *MITF*; ATAGGTGCATTGGCTTCTGG and TCTTCACCATGCTTTTGTGG for *TYR*; CATTTCCAGCTGGGTTTCTC and TGGTCTGTGAATCCTTGGAA for *TYRP1*.

### 4.6. Cellular Tyrosinase Activity Assay

Cellular tyrosinase activity was determined using a previously described method with modification [25,26]. Cultured B16F10 cells were incubated with α-MSH in the absence or presence of plumbagin, and cells were then washed and lysed with PBS containing 1% sodium deoxycholate and 0.5% Triton X-100. After the protein concentration was determined, 50 μg of protein was incubated with 2.0 mM l-DOPA and 0.1 M PBS (pH 6.8) for 1 h at 37 °C. The oxidation of l-DOPA was measured at 475 nm (OD_475_) using an absorbance reader. Activity was measured using the following formula: tyrosinase activity (%) = (OD_475_ of sample/OD_475_ of control) × 100.

### 4.7. Cell-Free Tyrosinase Activity Assay

To determine the inhibitory effect of plumbagin on tyrosinase activity, purified mushroom tyrosinase was incubated for 2 h with reaction mixture containing 0.1 M PBS (pH 6.8) and 2 mM l-DOPA in the absence or presence of plumbagin. The oxidation of l-DOPA to dopachrome was measured at 475 nm (OD_475_) with an absorbance reader. Activity was measured using the following formula: tyrosinase activity (%) = (OD_475_ of sample/OD_475_ of control) × 100.

### 4.8. DPPH Radical Scavenging Activity Assay

To determine the radical scavenging activity of plumbagin, a 0.2 mM DPPH solution in methanol was prepared and used. Each sample was diluted with distilled water to final concentrations of 0.1, 0.2, 0.5, 1, 2, and 5 mg/mL (plumbagin), or 0.005, 0.01, 0.05, 0.1, 0.2, and 0.5 mg/mL (vitamin C). After reaction, optical density (OD) was measured at 517 nm (OD_517_) using an absorbance reader. Free radical scavenging activity was calculated using the following formula: DPPH radical scavenging activity (%) = 10 − ((ODs/ODc) × 100), where ODs represents the absorbance of the samples, and ODc represents the absorbance of the vehicle control.

### 4.9. Statistical Analysis

All data were analyzed using an unpaired Student’s *t*-test for two experimental comparisons and a one-way analysis of variance (ANOVA) with Tukey’s post hoc test for multiple comparisons using Prism (GraphPad Software Inc., La Jolla, CA, USA). Data are presented as mean ± standard deviation (SD). Differences between mean values were considered statistically significant when the associated *p*-value was less than 0.05.

## 5. Conclusions

The major findings of this study are that plumbagin (i) suppresses α-MSH-induced melanin synthesis by inhibiting tyrosinase activity; (ii) does not affect the MITF-linked transcriptional machinery and signal transduction cascades including AKT and ERK1/2 activation associated with melanogenesis; and (iii) does not cause toxicity in normal keratinocytes (HaCaT) and lens epithelial (B3) cells when its concentration was less than 5 µM. Taken together, plumbagin abrogates α-MSH-induced melanin synthesis by inhibiting tyrosinase activity independent of the CREB-MITF transcriptional axis. These results therefore suggest a promising and safe natural product plumbagin for decreasing hyperpigmentation by using it in the development of skin-whitening cosmetics.

## Abbreviations

α-MSH	alpha-melanocyte stimulating hormone
TYR	tyrosinase
l-DOPA	l-3,4-dihydroxyphenylalanine
MITF	microphthalmia-associated transcription factor
TYRP1	tyrosinase-related protein 1
CREB	cAMP response element binding protein
PKA	protein kinase A
ERK1/2	extracellular signal-regulated kinase 1/2
